# Increasing Damping of Thin-Walled Structures Using Additively Manufactured Vibration Eliminators

**DOI:** 10.3390/ma13092125

**Published:** 2020-05-03

**Authors:** Paweł Dunaj, Stefan Berczyński, Karol Miądlicki, Izabela Irska, Beata Niesterowicz

**Affiliations:** Department of Mechanical Engineering and Mechatronics, West Pomeranian University of Technology, Szczecin, al. Piastów 19, 70-310 Szczecin, Poland; stefan.berczynski@zut.edu.pl (S.B.); karol.miadlicki@zut.edu.pl (K.M.); izabela.irska@zut.edu.pl (I.I.); beata.niesterowicz@zut.edu.pl (B.N.)

**Keywords:** thin-walled structures, vibration eliminators, additively manufactured parts, finite element method, composite

## Abstract

The paper presents a new way to conduct passive elimination of vibrations consisting of covering elements of structures with low dynamic stiffness with polylactide (PLA). The PLA cover was created in 3D printing technology. The PLA cover was connected with the structure by means of a press connection. Appropriate arrangement of the PLA cover allows us to significantly increase the dissipation properties of the structure. The paper presents parametric analyses of the influence of the thickness of the cover and its distribution on the increase of the dissipation properties of the structure. Both analyses were carried out using finite element models (FEM). The effectiveness of the proposed method of increasing damping and the accuracy of the developed FEM models was verified by experimental studies. As a result, it has been proven that the developed FEM model of a free-free steel beam covered with polylactide enables the mapping of resonance frequencies at a level not exceeding 0.6% of relative error. Therefore, on its basis, it is possible to determine the parameters of the PLA cover. Comparing a free-free steel beam without cover with its PLA-covered counterpart, a reduction in the amplitude levels of the receptance function was achieved by up to 90%. The solution was validated for a steel frame for which a 37% decrease in the amplitude of the receptance function was obtained.

## 1. Introduction

Structural damping largely determines the dynamic properties of mechanical structures especially those whose functioning is accompanied by time-varying loads. These loads may cause vibrations of a different nature, which adversely affects the functionality of the structure. Therefore, engineers are constantly looking for solutions to reduce vibrations using active and passive vibration elimination methods [1,2,3,4,5]. Active methods of vibration elimination consist of introducing an additional system into the mechanical system, which enables the elimination of vibrations by compensating them from additional sources. These methods are used in industries such as the machine tool industry [6,7,8,9,10], automotive industry [11,12], and aviation industry [13].

Passive vibration elimination methods consist in modifying the parameters and structure of mechanical systems in such a way as to increase their ability to dissipate vibration energy. Recent studies have shown that polymeric materials give promising results related to the ability of these materials to dissipate vibration energy [14]. Therefore, they are increasingly used in the passive elimination of vibrations.

In References [15,16], authors presented a solution that consisted of applying a thin layer of epoxy system to the foundation of the linear guide system of the milling machine. The presented results of dynamic properties tests showed that this solution improves the damping properties of the guide system in question. An almost two-fold decrease in the amplitude of the receptance function for selected resonance frequencies was obtained. The disadvantage of the presented solution is a significant loss of static stiffness for larger layer thicknesses of the epoxy resin.

The authors in References [17,18,19] present a method of increasing the capacity of steel structures to dissipate vibrations by using a polymer concrete infill. The authors presented the main body of a vertical lathe made of hollow section steel profiles, connected to one another by welded joints. The inner space of these profiles was filled with polymer concrete, which consists of epoxy resin and a mineral filler of various sizes. The main advantage of this solution was the possibility of shaping the dynamic properties of the body by means of an appropriate arrangement of the infill material, which ensures the possibility of local increase in weight, stiffness, and damping of body components. The authors showed that the use of polymer concrete infill makes it possible to reduce the amplitude of the receptance function by up to 85%. However, the disadvantage of the presented solution is a significant increase in the weight of the structure, which significantly limits its scope of application.

In the paper [20], the authors conducted experimental and numerical research on the composite support beam. The beam consisted of two aluminium bars connected together using polymer adhesive with a high damping coefficient. The analyses were carried out to determine the dynamic properties of the beam for different thicknesses of the adhesive layer, i.e., to determine the values of natural frequencies and the corresponding form of natural frequencies and damping coefficients. Comparing the results of tests for composite beams with those obtained for aluminium beams, it has been proven that, when using a layer of polymeric adhesive, it is possible to achieve even more than a ten-fold increase in the damping coefficient.

In Reference [21], the author analyses the effectiveness of damping resonance vibrations of a spring using a new method of local coatings made of highly-damping material covering its last coils (five coils at each end of the spring) as well as the influence of these coatings on the maximum values of dynamic stresses and the values of natural vibration frequencies of springs. The author shows that, while an elastomeric coating applied on the whole length of spring wire always causes a decrease in the first natural frequency of the spring, an application of the same amount of damping material only on its end-coils may lead to an increase in this frequency. The author derived a mathematical model that allows the calculation of the effectiveness of dynamic stress reduction both in the spring and the coating itself, for arbitrary geometrical and material properties of coatings. The numerical and experimental examples showed that, thanks to the coating used, the stress amplitudes in the spring can be reduced by more than three times.

An alternative way to increase vibration damping is to use additively manufactured polymer materials. In Reference [22], the authors presented an extendable multi-material projection microstereolithography process capable of producing carbon-fibre-reinforced cellular materials that simultaneously achieve high specific stiffness and a damping coefficient. Inspired by the upper bounds of stiffness-loss coefficient in a two-phase composite, the authors designed and additively manufactured carbon fiber reinforced polymer (CFRP) micro-lattices with soft phases shaped into selected stiff-phase struts. The results of both experimental and analytical calculations revealed that the damping performance can be significantly enhanced by the addition of only a small fraction of the soft phase. The presented design and additive manufacturing strategy allow for optimizing mutually exclusive properties. As a result, these CFRP micro-lattices achieved high specific stiffness comparable to commercial CFRP, technical ceramics, and composites, while being dissipative like elastomers.

This paper presents a new method of passive elimination of thin-walled structure vibrations consisting of the application of covers made of polylactide with a high damping capacity manufactured using the 3D printing technology. The increase in damping of thin-walled structures is achieved by the interference fit of the polymer covers with the structure elements. In this paper, experimental studies in the form of a three-point bending test and impact test were conducted in order to determine the values of material properties of the applied polylactide. In order to determine the shape, thickness, and distribution of the polylactide covers, a parametric analysis was carried out, using variants of the listed properties. This analysis was carried out using a finite element model, on the example of a steel beam with a square closed section of 70 mm × 70 mm, a wall thickness of 3 mm, and a length of 1000 mm, which was covered with polylactide. Lastly, the example of an application of presented eliminators to increase damping of the steel frame was shown. The proposed method enables even a 90% reduction for a beam and 37% reduction for a frame of the amplitude of the receptance function for resonance frequencies with a minimum increase in the mass of the structure and unchanged stiffness. Additionally, due to its passive character, the applied method does not require the use of complex measuring and execution systems.

The structure of the paper is as follows. In Section 2, the concept of additively manufactured vibration eliminators is presented. Next, experimental studies to determine the static and dynamic properties of the analysed eliminators are conducted. In Section 3, a finite element model of the free-free steel beam with polylactide (PLA) eliminators is established and the parametric analyses of the influence of the thickness of the cover and its distribution on the increase of the dissipation properties of the steel beam are conducted. Next, the numerical results are verified experimentally and validated on the basis of steel frame analysis. In Section 4, discussion of the results obtained was carried out. Section 5 contains the final conclusions that summarize the most important achievements of the article.

## 2. Materials and Methods

### 2.1. Concept of Additively Manufactured Vibration Eliminators

The concept of vibration damping presented in this paper is based on the use of special polymer covers to increase the structure’s ability to dampen vibrations. Polymers show very good damping properties, which reduce vibrations and isolates noise, shocks, and impacts [23]. Polymers and polymer composites are usually characterized by a high loss factor η in comparison with metals (in plastics, the loss factor is in the order of 1–200%, in metals, it is in the order of 0.1–1%) [23]. Materials for applications in polymeric covers should be characterized by damping capacity in the frequency ranges required for a given application as well as high mechanical strength and good thermal stability. In this particular case, the requirements for structural stiffness force the selection of materials with high strength parameters and stability at elevated temperatures of the structure operation. Such requirements exclude the use of elastomeric pads (rubbers, polyurethane elastomers, thermoplastic elastomers), which show excellent damping properties but are characterized by low mechanical and thermal strength. A good solution seems to be to use classical, thermoplastic structural materials/high-temperature, bio-based, and/or biodegradable materials instead. Currently, thermoplastics for fused deposition modelling (FDM) printing technology available on the market include: acrylonitrile butadiene styrene (ABS), polycarbonate (PC), polyamide (PA), polyphenylsulphones (PPS/PSU), polyetherimide (PEI), polyether ether ketone (PEEK), and polylactide (PLA) [24,25,26].

Of these, the last one mentioned attracts the greatest interest. This is determined by a number of advantages, which include good mechanical properties, ease of processing, and a low price. PLA obtained from renewable sources belongs to the group of aliphatic thermoplastic polyesters. The mechanical and thermal properties as well as the biodegradability of PLA depend, to a large extent, on its molecular weight and the proportions of individual lactide stereoisomers. Depending on the type, PLA is characterized by a melting temperature (T_m_) of 160–240 °C, glass transition temperature (T_g_) of 55–60 °C, and tensile strength from 30 to 50 MPa [27,28,29]. An unquestionable advantage is the fact that PLA does not require high temperatures during processing. Usually, the plasticization temperature varies between 160–200 °C [30]. Moreover, PLA as one of the few plastics does not require a heated working table. Furthermore, polylactide is a slow crystallizing material [31]. The cooling process during processing using the FDM method effectively prevents its crystallization. Thus, models made of PLA are characterized by a low processing shrinkage (< 0.5%), high dimensional accuracy, and shape stability [32]. Taking into account the presented advantages of PLA, it is necessary to use this material for further analyses.

**Figure 1:**
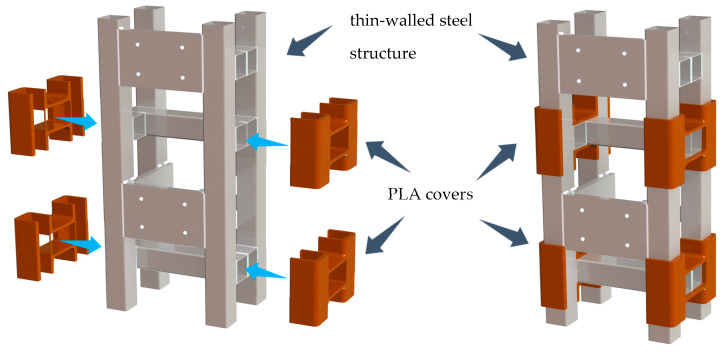
Concept of additively manufactured eliminators.

To be more precise, the increase in damping of mechanical structures is achieved by the interference fit of the polymer covers with the structural elements. The covers used have been manufactured using 3D printing technology. The concept of eliminators produced in the form of elements printed from PLA is presented in Figure 1.

The application of the proposed solution brings a number of advantages including:

- the ability to effectively increase the damping of new and existing mechanical structures;
- possibility of easy modification of parameters of the mass-dissipation-elastic system;
- the use of a material such as PLA is economically justified;
- the applied material is fully biodegradable;
- the application of 3D printing, for example fused deposition modeling (FDM), eliminates the need to produce tools (e.g., injection moulds) and design a complex technological process;
- when the cover is worn, it can be replaced quickly and easily;
- the method of fixing the damping covers using interference fit (e.g., j7/H6) excludes the need to interfere with the structure or the use of intermediary elements or bonding agents (e.g., adhesives).

### 2.2. Samples Preparation

**Figure 2:**
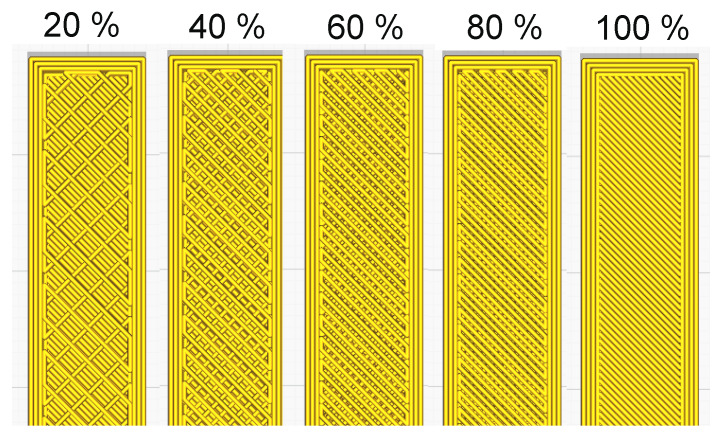
View of the Zig Zag pattern depending on the percentage of infill.

PLA samples for three-point bending tests in the form of 80 mm × 10 mm × 4 mm cubes were tested according to ISO178-2010 [33]. The analyzed samples were manufactured using injection moulding and 3D printing methods. Samples made using the FDM method were produced with the application of the i3 Mega 3D printer (Anycubic, Shenzhen, China), with the following printing parameters: plasticization temperature: 205 °C, table temperature: 60 °C, ambient temperature: 23 °C, nozzle diameter: 0.4 mm, layer thickness: 0.2 mm, model orientation: Zig Zag, printing speed: 50 mm/s, wall thickness: 1.2 mm, wall line count: 3, and top/bottom layers: 6. Samples were made using Ecoline PLA filament (Print-Me, Gorzów Wielkopolski, Poland) with a thickness of 1.75 mm. The samples were produced in five variants differing in infill density (20%, 40%, 60%, 80%, and 100%) (Figure 2). The G-code for printing was generated using Cura 4.5 software (Ultimaker, Geldermalsen, Netherlands).

In order to obtain samples using injection moulding, the filament was granulated, dried in-line for 24 h, and processed using a Dr. Boy GmbH & Co. injection moulding machine with a clamping force of 15 tons (Neustadt-Fernthal, Germany). The injection pressure was about 50 MPa, the contact pressure was about 41 MPa, and the temperature of the plasticizing system was 15 °C higher than the PLA melting temperature.

The specimens prepared in this way were conditioned at room temperature for at least two weeks after the processing (3D print/injection moulding). The specimen for an impulse test (a cuboid sample of 30 mm × 30 mm × 250 mm, with 60% infill) was prepared in an analogous manner.

### 2.3. Static Tests

The three-point bending tests were performed on Autograph AG-X plus (Shimadzu, Tokyo, Japan) equipped with a force sensor with a measuring range up to 1 kN (1.0 class). The crosshead speed was set to v_c_ = 1 mm/min. The values of Young’s modulus were determined from the linear range of the stress-strain curve (for deformations of 0.0005–0.0025). For each infill, seven measurements were taken. The mean and standard deviation for Young’s modulus, flexural stress, and strain were determined.

The density of PLA was determined using a hydrostatic method with distilled water at 23 °C as the immersion liquid. The measurements were carried out with Radwag AS160 C2 (Radom, Poland) laboratory balance cooperating with the kit for determining solids’ density.

### 2.4. Dynamic Tests

**Figure 3:**
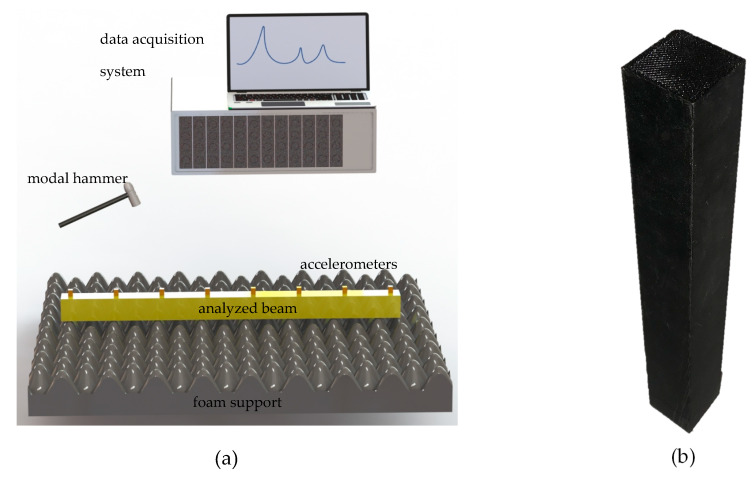
Diagram of the test stand for determining the loss factor (**a**) and analyzed beam (**b**).

Next, dynamic tests were carried out to determine the loss factor of the material concerned. The tested specimen (a cuboid beam of 30 mm × 30 mm × 250 mm) was supported on a special foam, which minimized the influence of fixing on the dynamic properties of the tested specimen. Excitation was carried out in the middle of the beam using a PCB 086C01 modal hammer (PCB Piezotronics, Depew, NY, USA). The response of the tested specimen was measured at eight points evenly spaced along the length of the beam using PCB 356A01 sensors (PCB Piezotronics, Depew, NY, USA). Frequency response functions were determined in a direction perpendicular to the surface to which the accelerometers were attached (Figure 3). Signal acquisition was performed using a Scadas Mobile Vibco analyzer (Siemens, Munich, Germany) and LMS Testlab 2019.1 software (Siemens, Munich, Germany). The parameters of the signal acquisition were presented in Table 1. Signal acquisition time was set on 2 s to ensure vibration amplitude decrease below the noise level, which eliminates the need for windowing response signals. Estimation of the frequency response functions was performed using an H1 type estimator. In order to minimize the influence of random and systematic errors on estimated FRFs, their estimation was made on the basis of averaging from 10 forcing implementations. The value of the loss factor was determined as the average value of the first (dominant) resonance calculated from eight measurement points. Loss factor for each point was determined according to Reference [34]. The value of the loss factor determined was shown in Table 3.

### 2.5. Experimental Test Results

**Figure 4:**
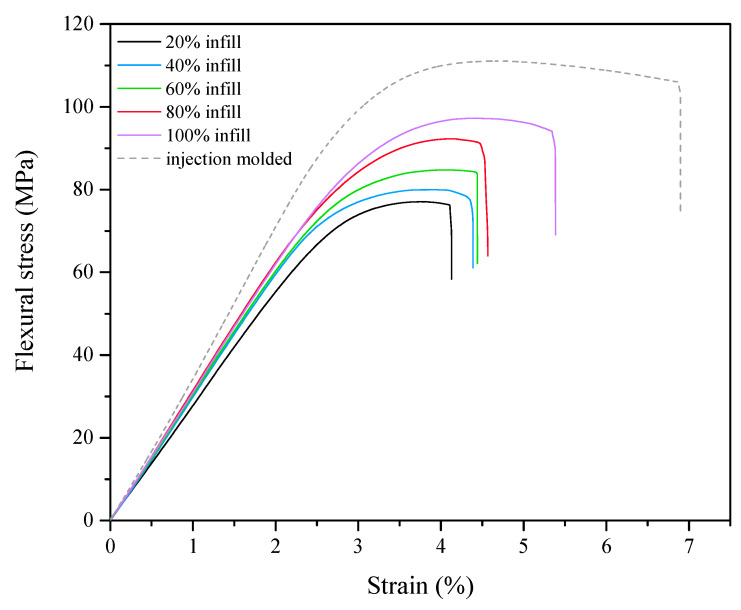
A stress-strain diagram for the three-point bending test.

The results obtained for printed specimens were compared with characteristic values for specimens produced by injection moulding. Representative stress-strain graphs determined in the bending test are shown in Figure 4, whereas the numerical values with standard deviations are shown in Table 2.

As could be predicted, the samples produced by the additive manufacturing method are characterized by slightly reduced mechanical properties compared to the injection moulded samples. The values of Young’s modulus, flexural stress, and flexural strain for samples produced by 3D printing with maximum infill (100% infill) are lower, respectively, by: 9.31%, 12.42%, and 21.73%, relative to injection moulded samples. Such a distribution of results can be explained by differences in macrostructure of samples obtained with the use of the mentioned techniques. The samples obtained by injection moulding are homogeneous, without pores and cavities, while the models made by 3D printing are characterized by a layered structure. In the latter method, the individual layers of plasticized material are adhesively bonded, which leads to weakening of the model’s strength at the points where the surfaces are joined [25,35]. On the basis of the presented results, it can be stated that, with the decrease of infill density, the strength properties of the samples produced by the FDM method gradually deteriorate. For samples with 20% infill, the recorded values of Young’s modulus, bending stress, strain at max. stress, and strain at fracture were reduced by ca. 21%, 30%, 19%, and 40%, respectively, as compared to reference samples (produced by injection moulding). Although the details produced by the 3D printing method exhibit inferior mechanical properties, the variation over the tested infill range is not very significant. Moreover, it has to be said that the polymeric pads are neither vital for the construction itself nor subjected to high mechanical loads when operated. Therefore, the choice of infill for this particular application is quite open. Herein, the 60% infill was chosen for both finite element modeling and experimental tests, as a compromise between sufficient mechanical properties, cover weight, and printing time.

As a result of the dynamic tests, eight frequency response functions were determined, which, in turn, were used to calculate the value of the loss factor. Due to the low modal density, the half power method was used to determine the loss factor.

The values of PLA and steel (presented in order to build a steel beam model) material properties, supplemented by standard uncertainty values, are presented in Table 3. The determined values of material properties were used as input data for constructing the finite elements model (FEM) of a beam covered with a damping material.

## 3. Finite Element Modelling

### 3.1. Finite Element Model of a Free-Free Beam

In order to determine shape, thickness, and distribution of the polylactide covers a model of a beam covered with polylactide (60% infill) was built using the finite element method and the values of material properties determined in the previous section. On the basis of the built model, a parametric analysis of solutions differing in cover thickness was carried out. The aim of this analysis was to find a solution that would ensure a significant increase in the damping of the structure with a minimal increase in its mass.

The construction of the model began with the discretisation of a geometric model of a steel beam with a square closed section of 70 × 70, a wall thickness of 3 mm, and a length of 1000 mm. Discretisation was carried out using the Midas NFX 2018 R1 pre-processor (Midas Information Technology Co. Ltd., Seongnam, South Korea) [36]. In order to obtain the best possible accuracy of the model, a structural lattice made of isoparametric, eight-node, cubic finite elements of CHEXA type was used.

**Figure 5:**
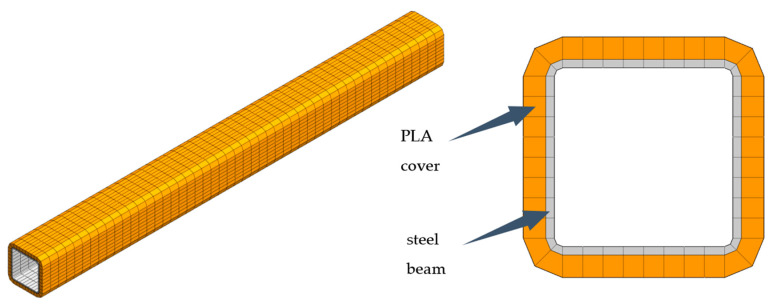
Finite element model of a polylactide-covered beam.

Then, in an analogous way, the polylactide cover was discretised. The steel beam and the polylactide cover were connected to each other using nodes coincidence. The model consisted of 4000 finite elements and had 18,360 degrees of freedom. The finite element model of a steel beam covered with polylactide is shown in Figure 5.

Next, the model describing the damping properties of the beam in question was selected. A structural damping model was used, according to which the damping matrix C can be expressed as:

(1) C=iηK

where: K—model stiffness matrix, i—imaginary unit, η—loss factor.

### 3.2. Influence of PLA Cover Thickness on Damping Abilities

**Figure 6:**
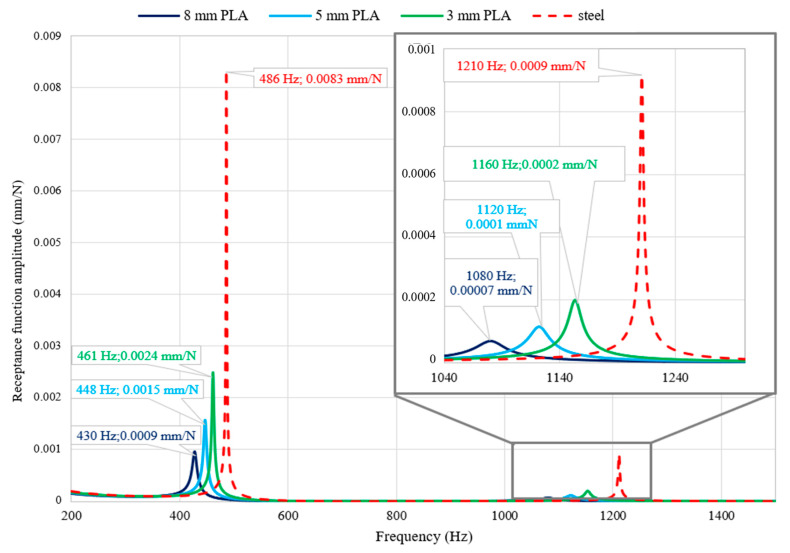
Comparison of the receptance functions for steel beam with the applied polylactide (PLA) cover of different thicknesses.

Three models differing in the thickness of polylactide cover were built. In the analysis, 3-mm, 5-mm, and 10-mm thick cover were used, respectively. For the unconstrained (free-free) models receptance functions [mm/N] in the range of 10–1500 Hz were determined. The excitation point was 400 mm from the end of the beam, while the response measurement point was 200 mm from the end. The receptance functions were determined in a direction perpendicular to the upper face of the steel part of the beam analyzed. The calculations were performed using the Nastran Solver 2018 R1 (SOL108). The comparison of the receptance function for the selected point is shown in Table 4 and Figure 6.

Analysing the receptance functions shown in Figure 6, it can be seen that the use of polylactide cover significantly increases damping, which allows up to a 90% amplitude reduction for resonance frequencies. As the thickness of the polylactide cover increases, the structure’s ability to absorb vibrations is increased. This phenomenon can be seen in both the first and the second resonance frequency. Additionally, as the thickness of the polylactide cover increases, the resonant frequencies decrease.

### 3.3. Influence of PLA Cover Placement on Damping Abilities

However, the use of a damping material over its entire length means a significant increase in the weight of the structure. Given the objective outlined previously, it was decided to carry out an analysis for a discontinuously covered beam. It was decided that the beam would be covered with polylactide in the characteristic points of the mode shapes, i.e., points with maximum and minimum value of vibration amplitude.

In order to determine those points, the eigenvalue problem was solved.

(2) $$\left(K-\omega_{j}{}^{2}M\right)\Phi_{j}=0$$

where ***M***—mass matrix, $\omega_{j}$—j-th natural frequency, $\Phi_{j}$—j-th mode shape vector.

**Figure 7:**
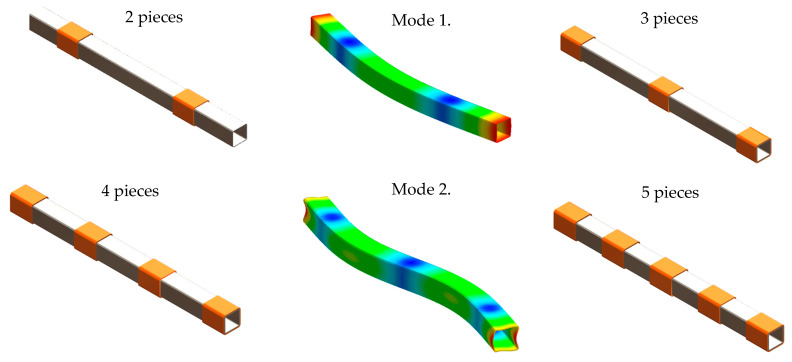
Mode shapes of steel beam and variants with different locations of the PLA covers subjected to further analysis.

The calculations related to solving the eigenvalue problem were performed using Midas NFX 2018 R1 (SOL103) software. Then, the distribution of the polylactide covers was determined on the basis of the obtained mode shapes of the steel beam. Thus, five variants were defined and analysed further. The variants were selected in such a way that the PLA covers were placed in points with maximum and minimum value of vibration amplitude of the first two mode shapes of steel beam. A PLA cover thickness of 8 mm and 100 mm long was assumed for the analysis. Figure 7 shows the mode shapes of the steel beam and selected variants, which was subjected to further analysis.

**Figure 8:**
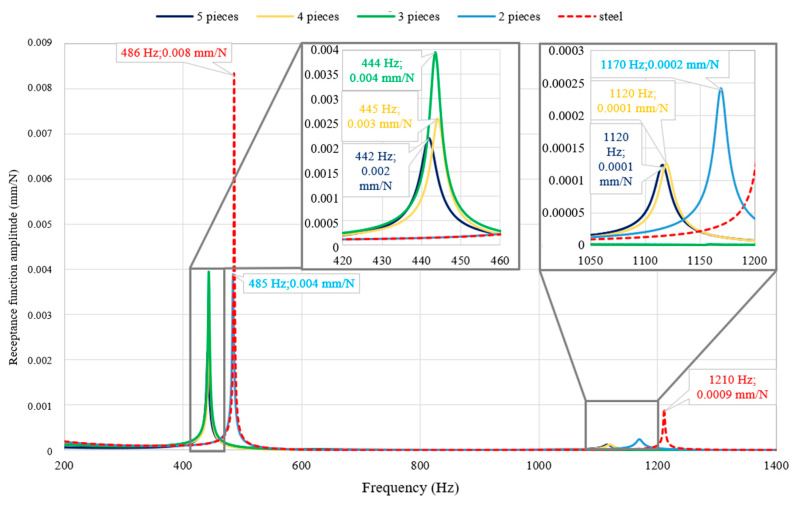
Comparison of the receptance functions for the partially covered beam with a different covers location.

Receptance functions obtained as a result of FEM model solution are presented in Table 5 and Figure 8.

Analysing the obtained receptance functions, it can be seen that the use of a partial polylactide cover increases damping. In addition, the position of the damping pads is decisive in terms of vibration damping for a specific resonance frequency. When a polylactide cover consisting of three parts is used, the phenomenon of the mode shape disturbance and the related disappearance of the second resonant frequency occurs. To sum up, it can be stated that a partial covering of steel beam with polylactide can satisfactorily increase its damping.

### 3.4. Experimental Verification of Selected Solution

The solution characterized by the best damping (five pieces) was experimentally verified. The tests were carried out using the impulse test with the use of the stand described in Section 2.4. In order to best reflect the free-free constraints, the tested beam was suspended on steel cables. The PLA covers were attached to the steel beam using tight fit (j7/H6).

**Figure 9:**
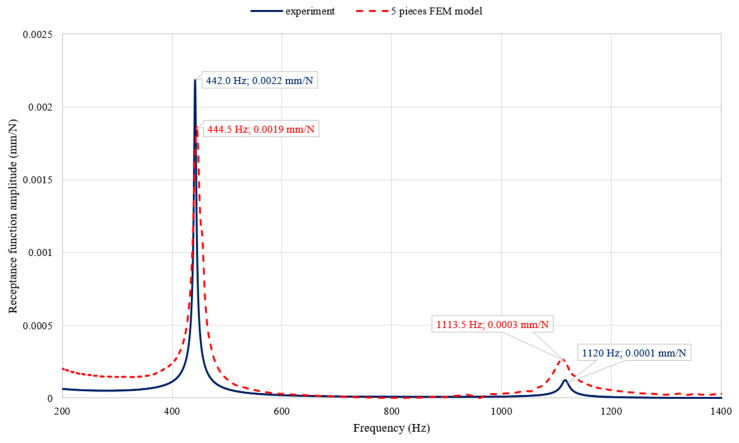
Comparison of the selected receptance function determined by calculation and experimentally.

The comparison of the selected receptance function determined on the basis of the model analysis with the experimental results is shown in Figure 9.

Analysing the results obtained, it was noted that the proposed FEM model reliably reproduced the dynamic properties of the steel beam in question with PLA cover.

As a measure of the compatibility of resonant frequencies of the FEM model of the considered beam with the resonant frequencies determined experimentally, the value of relative error δ was assumed, which is defined as follows.

(3) $$\delta =\left|\frac{f_{exp}-f_{FEM}}{f_{exp}}\right|\cdot 100\%$$

where $f_{exp}$—experimentally determined resonant frequency and $f_{FEM}$—resonant frequency determined on the basis of the FEM model.

As a result, it was found that, for both resonant frequencies, their values complied with a level not exceeding 0.6% of a relative error. The values of the amplitude of the receptance function for both resonances were reproduced with satisfactory accuracy.

### 3.5. Steel Frame Example

In order to prove the utilitarian character and, above all, the industrial applicability of the presented solution, an example of the application of partial/point polylactide cover to increase the vibration dissipation capacity of the steel frame is presented. Specifically, it was decided that the main objective would be to determine the position of the polylactide cover in such a way as to minimize the transverse beam vibrations.

The frame under consideration consisted of three steel beams with a closed square section of 70 mm × 70 mm, wall thickness of 3 mm, and lengths of 500 (vertical beams) and 1000 mm (transverse beam), respectively. The connection of individual beams was implemented by means of welds.

**Figure 10:**
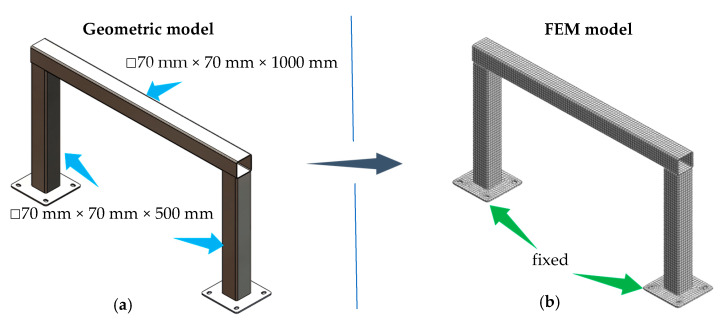
Geometric model (**a**) and discrete model (**b**) of the steel frame under consideration.

In the first stage, the FEM model of the considered frame was built. The model consisted of 7481 finite elements and 12,947 degrees of freedom. A structural mesh consisting of CHEXA elements was used to build the model. Then, for the model discretised, boundary conditions were determined by taking the degrees of freedom, according to Figure 10.

**Figure 11:**
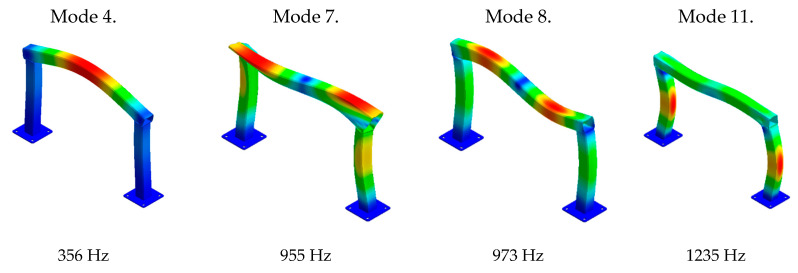
Steel frame mode shapes characterized by transverse beam vibrations.

A modal analysis was conducted for the model. As a result, values of natural frequencies (Table 6) and corresponding mode shapes were obtained. Analysing the results obtained, the mode shapes characterized by increased transverse beam vibrations were identified. These forms are shown in Figure 11.

**Figure 12:**
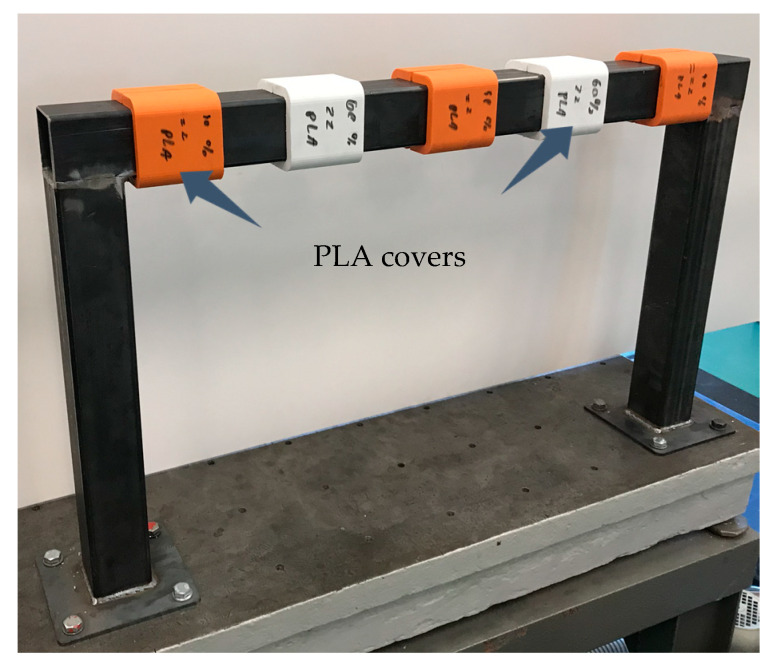
Distribution of the PLA covers.

The distribution of PLA covers was determined by analysing the obtained mode shapes. It was decided that the polylactide covers will be placed in points with maximum and minimum vibration amplitude of mode shapes for which obtaining increased damping was a priority. The distribution of the polylactide covers is shown in Figure 12.

**Figure 13:**
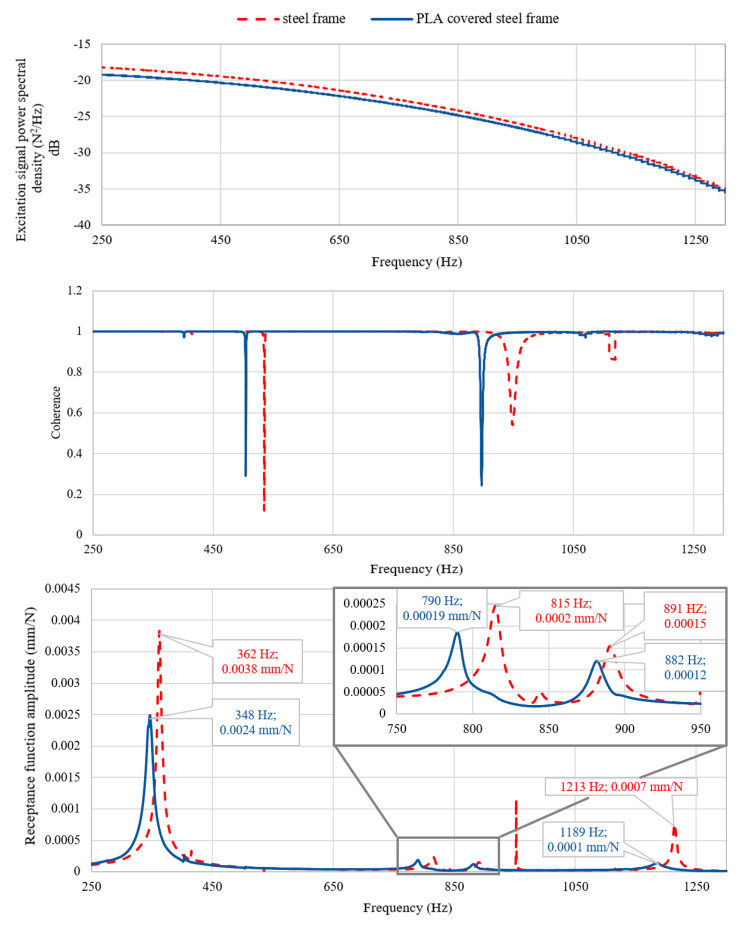
Comparison of receptance functions for steel frame and a steel frame with PLA covers.

Then, for a steel frame and a frame with polylactide covers distributed in accordance with Figure 12 and attached using tight fit j7/H6, the frequency response functions were experimentally determined using a test stand presented in Figure 3. Estimation of the frequency response functions was performed using an H1 type estimator. The excitation point was 400 mm from the end of the beam, while the response measurement point was 600 mm from the end. The receptance functions were determined in a direction perpendicular to the upper face of a transverse beam. Receptance functions with corresponding coherence functions, and power spectral density function of excitation determined experimentally for steel frame and a frame with PLA covers are compared in Figure 13.

## 4. Discussion

Having analysed the results obtained, it was found that the application of polylactide cover results in a significant increase in the steel structure’s ability to damp vibrations. Therefore, the following detailed conclusions can be drawn.

- In the case of a free-free steel beam, with an increase in the thickness of the polylactide cover, its ability to dampen vibrations increases. Thus, for the first resonance, a 71% decrease in the amplitude of the receptance function was obtained for a 3-mm thick cover, with an 82% decrease for 5 mm and a 90% decrease for 8 mm;
- The increase in the damping of the structure can also be achieved by covering it in a non-continuous manner. Therefore, a 50% decrease in amplitude for the first resonance is achieved for two and three elements, a 62.5% decrease is achieved for four elements, and a 75% decrease in amplitude is achieved for five elements;
- Comparing obtained results with those presented in Reference [17], it can be seen that presented method gives better results for the beams analysed. A 90% decrease in the amplitude of the receptance function compared to a 73% decrease in Reference [17]. Moreover, these results were achieved with the beam weight increased by 28%, while, in the case of Reference [17], the beam weight was increased by 114%;
- The use of non-continuous cover (three elements placed in the nodes of the second form of vibration) in the case of a free steel beam resulted in the appearance of a phenomenon known as a disturbance of the mode shapes, which resulted in the disappearance of the second resonance;
- The use of a polylactide cover to increase the damping of the steel frame resulted in a 37% decrease in the amplitude of the first resonance function. For the remaining resonances in the analysed frequency range, an average 37% decrease in amplitude was obtained;
- By analysing the accuracy of the mapping of the dynamic properties of a steel beam partially covered with polylactide (five elements), it can be seen that, for both resonant frequencies, a compliance of their values not exceeding 0.6% of relative error and a satisfactory compliance of the amplitude of the receptance function for the first mode was obtained.

## 5. Conclusions

To sum up, this paper presents a new way of passive elimination of vibrations of thin-walled structures with low dynamic stiffness consisting of covering the structure with polylactide. In addition, the paper formulates a methodology (based on modal analysis of the FEM model of the structure) for selecting the location of the covering in order to increase the damping for a specific form of vibration. The presented analysis results for both the free beam and the steel frame prove the effectiveness of the proposed method, which may become an alternative to the currently used passive vibration elimination methods, which was proven by comparing the obtained results with other research studies. The main limitation of the presented method is its effectiveness for increasing the damping of non-thin-walled structures.

## Figures and Tables

**Table 1:** Parameters of signal acquisition.

Parameter	Value
Sampling rate	4096 Hz
Frequency resolution	0.5 Hz
Signal acquisition time	2 s
Scaling of the frequency response function	global

**Table 2:** Flexural properties of 3D-printed/injection moulded PLA specimens.

Sample Code	Flexural Young’s Modulus (GPa)	Flexural Stress (MPa)	Deflection at Max. Stress (%)	Deflection at Break (%)
20% infill	2.63 ± 0.07	77.1 ± 1.1	3.8 ± 0.1	4.1 ± 0.3
40% infill	2.86 ± 0.13	80.0 ± 3.2	3.9 ± 0.1	4.4 ± 0.4
60% infill	2.91 ± 0.19	84.8 ± 5.0	4.0 ± 0.1	4.4 ± 0.4
80% infill	3.02 ± 0.10	92.3 ± 4.0	4.1 ± 0.3	4.6 ± 0.4
100% infill	3.02 ± 0.06	97.3 ± 2.0	4.4 ± 0.1	5.4 ± 0.4
Injection moulded polylactide (PLA)	3.33 ± 0.13	111.1 ± 0.7	4.7 ± 0.1	6.9 ± 0.6

**Table 3:** Material property values of PLA (60% infill) and steel.

Property	Polylactide	Steel [19]
Young’s modulus E	2.91 ± 0.19	210 ± 5 GPa
Density ρ	1.254 ± 0.003 g/cm^3^ *	7812 ± 35 kg/m^3^
Loss factor η	0.0400±0.0008	0.00220 ± 0.00005

*: value determined for PLA reference samples (obtained by injection moulding).

**Table 4:** Free-free beams receptance function amplitudes for the first two resonances.

Property	1st Resonance Amplitude (mm/N)	2nd Resonance Amplitude (mm/N)	Mass (kg)
Steel	0.0083	0.0009	6.27
3 mm	0.0024	0.0002	6.92
5 mm	0.0015	0.0001	7.35
8 mm	0.0009	0.00007	8.07

**Table 5:** Receptance function amplitudes for the first two resonances for partially covered beams.

Property	1st Resonance Amplitude (mm/N)	2nd Resonance Amplitude (mm/N)	Mass (kg)
Steel	0.0083	0.0009	6.27
2 pieces	0.0040	0.0002	6.54
3 pieces	0.0039	–	6.68
4 pieces	0.0031	0.0001	6.81
5 pieces	0.0022	0.0001	6.95

**Table 6:** Natural frequency values of the steel frame.

Mode Shape	Natural Frequency
1.	117 Hz
2.	167 Hz
3.	219 Hz
4.	356 Hz
5.	487 Hz
6.	542 Hz
7.	955 Hz
8.	973 Hz
9.	1078 Hz
10.	1219 Hz
11.	1235 Hz
